# The Effect of Ramadan Fasting on Biochemical and Performance Parameters in Collegiate Wrestlers

**Published:** 2012

**Authors:** Bahman Mirzaei, Farhad Rahmani-Nia, Mahdi Ghahremani Moghadam, Seyed Javad Ziyaolhagh, Abolfazl Rezaei

**Affiliations:** 1 *Faculty of Physical Education and Sport Sciences, University of Guilan, Rasht, Iran *; 2 *Department of Physical Education and Sports Sciences, Shahrood Branch, Islamic Azad University, Shahrood, Iran*; 3 *Faculty of Physical Education and Sport Sciences, Tehran University, Tehran, Iran*

**Keywords:** Body weight, Lipid profile, Performance, Power, Ramadan fasting

## Abstract

**Objective(s):**

This study was designed to evaluate the effect of Ramadan fasting on body composition, aerobic and anaerobic power, strength, plasma lipids profile and serum glucose among collegiate wrestlers.

**Materials and Methods:**

Fourteen male collegiate wrestlers (age, 20.12±2.5 yrs) volunteered as subjects for the study. Subjects were tested one week before the beginning of Ramadan, the last two days of Ramadan and the last two days of the 4th week after the end of Ramadan. The paired sample t-test was used to assess the differences in pre and post-performance tests and repeated measures ANOVA and Bonferroni *post hoc* test to determine differences between three blood samplings.

**Results:**

The results showed that except for anaerobic power and strength, body weight, body fat percentage and aerobic power at 4th week of Ramadan were significantly lower than pre-Ramadan values (*P=*0.05). Total cholesterol and low density lipoprotein levels decreased after Ramadan compared to pre-Ramadan (*P=* 0.011, *P=*0.001), however, a month after Ramadan, it reached to higher levels compared to pre-Ramadan period, which were not statistically significant. Similarly, significant decrease and increase were consequently observed in glucose and high density lipoprotein (*P=*0.001, *P=*0.045). Triacylglycerol and VLDL increased at the end of Ramadan compared to the period of time before Ramadan, and after Ramadan, it reached the lower levels compared to concentrations before Ramadan (*P=*0.133, *P=*0.133).

**Conclusion:**

This study also indicated that Ramadan fasting appears to have significant effect on body composition, aerobic power and lipid profile.

## Introduction

Ramadan is the holiest month in Muslims’ calendar. Fasting in this month is one of the main pillars of more than a billion Muslims among whom, more than 400 millions of them observe this divine. Ramadan takes place in different seasons and duration, from 8-16 hours in tropical countries. In this month, adult and healthy Muslims refrain from food, liquids and smoking from approximately 1 hr before sunrise till sunset, averaging about 15 hr a day for 29-30 days. Because of the type and duration of the diet, Ramadan is an excellent opportunity to study the effects of various diets on human body. 

There have been few studies on the effect of Ramadan fasting in athletes and especially in wrestlers, and this is still a matter of debate. Related studies showed that fasting in Ramadan without any health problems caused significant changes in body weight (1-6), blood glucose concentration (3, 5, 7) and lipid profile (3, 5, 8- 10). Moreover, sleep duration at night (4, 11, 12), and daily physical activity (3, 4, 13) were reduced during the month of Ramadan. These changes in diet and lifestyle have remarkable effects on the body metabolism (3). Some studies showed positive effects of fasting on the lipid profile and performance parameters (4 , 5, 9, 14), while some opposite effects were observed in others (3, 5, 6). 

Several studies have indicated that cardio-vascular responses (9, 15), biochemical metabolic changes and fat metabolism in Ramadan month, are related to the level of physical fitness and activity (2, 4). In addition, reduced metabolic rate at rest, dehydration (16) and hormonal changes (12, 17, 18) are other changes that have been reported in this month. However, significant reductions were reported in maximum oxygen consumption (VO_2 max_) in sedentary individuals (19, 20, 21) and footballers who (9, 22) followed a month of fasting. Some investigators (4) reported no influence of Ramadan fasting on aerobic, anaerobic power and capacity and also removal rate of lactate accumulation (LA) from blood following high intensity exercise. Additionally, they mentioned that if strength-power training is performed regularly and daily food intake, body fluid balance and daily sleeping time are maintained as before Ramadan, Ramadan fasting will not have adverse effects on body composition, anaerobic power and capacity, and LA metabolism during and after high intensity exercise in power athletes.

Although in recent years, there have been particular attentions to the effects of Ramadan fasting on various parameters involved in health, many of these studies were performed on untrained subjects and also few studies directly focused on athletes and especially on wrestlers. Competitive wrestling activity is extremely dynamic in nature and is one of the most physically and metabolically challenging events (23). Thus, the purpose of this study was the determination of Ramadan fasting effects on lipid profile, glucose concentration and performance parameters such as body composition, aerobic and anaerobic power and strength in elite collegiate wrestlers. 

## Materials and Methods

***Subjects***

Fourteen collegiate male wrestlers (age, 20.12 ± 2.5 yrs) volunteered as subjects for the study. They all had at least four years of training experience. The study received ethical approval from the Ethical Committee of Guilan University, Rasht, Iran.

***Study design***

Subjects were tested three times, one week before the beginning of Ramadan (Pre-RF), the last two days of Ramadan (End-RF) and the last two days of the 4th week after the end of Ramadan (After-RF). All performance measurements were taken at the same time of the day (between 10:00 a.m. and 12:00 a.m.) at a constant environmental temperature (22–25°C). Blood samples were also taken at the same time of the day (between 16:00 p.m. and 17:00 p.m. Within 48 hr prior to the resting, no intensive training was allowed. All subjects were at their preparatory training phase during the study. They maintained a similar training program (intensity, duration and frequency) to that of before Ramadan.

**Table 1:** Body composition and physical performance measurements (Mean ± SD) before and after Ramadan (n= 14)

Variables		Pre-RF	End-RF	t	*P* value
Body composition	Body mass (kg)	70.61 ± 18.40	68.96 ± 18.1	7.146	0.001*
	Fat free mass (kg)	60.28 ± 12.28	59.62 ± 12.14	2.63	0.019*
	Fat percentage (%)	13.39 ± 4.94	12.26 ± 5.07	4.03	0.001*
Aerobic power (ml.kg^-1^.min^-1^)	VO_2 max_	51.05 ± 5.90	49.19 ± 6.08	4.79	0.001*
Anaerobic power	Peak power (w)	675.19 ± 185.8	649.31 ± 175.76	1.53	0.145
	Mean power (w)	520.81 ± 113.30	489 ± 106.4	3.34	0.004*
	Fatigue index (%)	8.73 ± 3.44	8.67 ± 3.66	0.134	0.895
Strength	Squat (kg/w)	1.69 ± 0.36	1.75 ± 0.32	-1.838	0.086
	Bench press (kg/w)	1.31 ± 0.23	1.33 ± 0.22	-1.118	0.281
	Dead lift (kg/w)	1.54 ± 0.204	1.58 ± 0.20	-1.978	0.067

* Significant at α <0.05

***Performance parameters***

Body composition was assessed using a bio impedance method (inbody, 3.0). Measurements were performed without accessories that contain metal (rings, belts, coins). To ensure normal hydration status for BIA testing, participants were asked to adhere to the following pretest requirements: (a) no vigorous exercise within 48 hr prior to the test, (b) no caffeine or alcohol consumption within 12 hr prior to the test. Height and weight were also recorded.

For aerobic power assay, each subject performed a graded treadmill exercise test (Bruce protocol) in order to estimate VO_2 max_. In this study anaerobic power was measured by RAST (Running Anaerobic Sprint Test) protocol (6 × 35 m sprint with 10 sec rest intervals between sets) (24). Muscular strength tests included squat, bench press and dead lift. Venous blood samples were taken at the same time and before the physiological tests. 

***Statistical analysis***

General characteristics of the participants were presented as means and standard deviations. Paired sample t-test was used to assess the differences in pre and post-performance tests and repeated measures ANOVA (repeated measures factorial 2×3) and Bonferroni *post hoc *test were used to determine differences between three blood samplings. Level of significance was accepted at *P*<0.05.

## Results

Body composition (weight, free fat mass &body fat percentage), muscular strength (squat, bench press and dead lift), aerobic and anaerobic power measures are listed in Table 1. Results of Paired-sample t-test indicated that there were no significant changes in anaerobic power and strength of wrestlers before and after RF. Significant decreases were found in body composition and aerobic power between Pre-RF and End-RF.

As shown in Table 2, significant decrease and increase were consequently observed in glucose and HDL-C. Also, CH and LDL-C levels decreased after-RF comparing to pre-RF, however, one month after Ramadan (after-RF) it reached to higher levels comparing to pre-RF, which were not statistically significant. TG and VLDL levels in end-RF comparing to before-RF increased and after-RF gains lower levels comparing to levels in before-RF.

## Discussion

The present results showed that Ramadan fasting led to a decrease in body mass and body fat percentage. The results dealing with the effects of Ramadan fasting on anthropometric variables were inconsistent with the previous studies. 

According to Ramadan (2002) and Karli *et al* (2007), there were no significant changes on anthropometric variables during the Ramadan (4, 19). In contrast, Sweileh *et al* (1992), Ziaee *et al* (2006), Chaouachi *et al* (2008) and Haghdoost and Poorranjbar (2009) observed significant decrease in BW, BMI and BF%.( (3, 6, 21, 25).

**Table 2:** Plasma glucose, lipids and lipoprotein levels (Mean±SD) before, at the end and after Ramadan (n= 14)

	Pre-RF	End-RF	After-RF	*P-* value
Total cholesterol (mg/dl)	169.42 ± 22.02	154.92 ± 26.37	159.75 ± 25.90	0.011*
Triacylglycerol (mg/dl)	86 ± 19.75	89 ± 29.37	75.17 ± 24.34	0.133
HDL Cholesterol (mg/dl)	51.51 ± 10.84	53.54 ± 9.09	59.10 ± 4.90	0.045*
LDL Cholesterol (mg/dl)	100.20 ± 20.91	80.40 ± 24.25	87.07 ± 25.46	0.001*
VLDL Cholesterol (mg/dl)	17.20 ± 3.95	17.80 ± 5.87	15.03 ± 4.87	0.133
Glucose (mg/dl)	98.08 ± 2.94	82.67 ± 6.88	79.33 ± 6.05	0.001*

*Significant at α <0.05

Moreover, Frost and Pirani (1987) reported that Ramadan fasting caused significant increase in BW (26). The main possible factor when considering the reasons for this reduction may be the change in energy intake and hormonal alternations during the fasting period. As we will report later, decreases in glucose levels in this month occurs, which logically decrease the insulin levels and this consequences lead to enhance in fat oxidation. Also, considering that the subjects were wrestlers and that they kept their training program, more fat oxidation may occur. In particular, one might anticipate a depletion of both blood glucose and glycogen reserves, and thus an increased utilization of fat and a decreased utilization of carbohydrate during endurance exercise (27). Furthermore, part of this decline may be related to changes in body water levels.

The other finding in this study showed significant reduction in aerobic power. This suggests that during Ramadan the down regulation of body metabolism in the period of day time occurs, which reflect a conserving energy mechanism. Inhibition in catecholamine and reduced venous return has also been associated with fasting, causing a decrease in sympathetic tone, which consequently lead to a decrease in blood pressure, heart rate, and cardiac output (28, 29). These physiological variations may also affect performance capacity and cause downfall in athletes’ aerobic power (10). Sleeping patterns may also contribute to the significant decrease in athletic performance (12, 30). No significant difference was observed between Pre-RF and End-RF in strength and anaerobic power which corresponds to Gutierrez *et al *study (2001)*.* It seems that slight enhancement observed in strength was simply a reflection of reductions in body mass, as we calculated relative strength in this study. Karli *et al* (2007) suggested that if strength power training is performed regularly and daily food intake, body fluid balance and daily sleeping time are maintained as before Ramadan, Ramadan fasting will not have adverse effects on anaerobic power and capacity, and on LA metabolism during and after high intensity exercise in power athletes (such as wrestling). Wrestlers in this study performed their trainings as before Ramadan.

Similar to Ziaee *et al* (2006) and Azizi (1996) studies (6, 32), we observed a decrease in blood glucose, however, Mansi (2007), reported slight increase (5). Surprisingly, in this study we observed more decrease in glucose levels one month after Ramadan. It can be related to the metabolic adaptation during periods of Ramadan or it may also be related to the fact that wrestlers training did not allowe glucose’s maintenance and super compensation. Furthermore, changes in insulin levels can affect this decline. Farooq *et al* (2006) reported an increase in glucose-6-phosphatase (22 %) levels after Ramadan type fasting and this adaptation may occur in wrestlers in this study which is due to the decrease in glucose levels (33). 

Many reports have been published on the effect of Ramadan fasting on blood lipids among healthy individuals, with inconsistent and even conflicting findings. The discrepancy might be attributed to the amount and type of food intake, physical activity, ethnic, and genetic background of studied populations (9). In line with the reports of Ramadan *et al* (2002) and Mansi (2007 ((19, 5) we found a significant increase in serum HDL-C after Ramadan. The enhancement in serum HDL-C can be explained by either changes in fat intake or inherent metabolic changes during Ramadan. We also showed significant reduction in serum LDL-C and CH which is possibly due to the increase in fat oxidation induced by RF in trained athletes following submaximal exercises. The increased fat utilization may be related to reductions in body mass and body fat content. Similar to the Ziaee *et al* (2006) (6), serum TG and VLDL-C had not any significant changes in our study. The serum lipid profiles in healthy fasting individuals are considerably altered during Ramadan. The impact of fasting on the body metabolism is very complicated; many pathways alter to keep the body in balance. Physical activity, (15, 33) diet, (25, 27, 34) and sleeping hr (14, 35) change the body metabolism substantially. 

During Ramadan, all of these three main factors are changing (13, 36) and it is difficult to obtain a deep and comprehensive view on the impact of each factor alone. Most papers reported the impact of fasting during Ramadan in observational studies (1, 6, 9, 14). 

## Conclusions

Ramadan fasting appears to have significant effect on body composition, aerobic power and lipid profile. Ramadan fasting may be a healthy non-pharmacological method for improving lipid profile. Anyway, Ramadan fasting had no adverse effect on the subjects of the study. Athletes can save their physical capacity and sport performance with delicacy choosing training type and volume, daily caloric intake and food type, body fluid and electrolytic balance and adequate sleeping hours.
